# Rice Pudding

**Published:** 1886-01

**Authors:** 


**Rice Pudding.**—Cold rice pudding may be prepared and reserved at dinner by pouring a custard and a few turns of jelly or preserved fruit over it. Remember to remove the baked coating of the pudding before pouring the custard over it.

